# Postprocedure AntiBiotic prophylaXis For caRdiac Electrical devicE Implantation (ABxFREE): A Randomized Controlled Trial

**DOI:** 10.1016/j.jacasi.2025.06.001

**Published:** 2025-07-29

**Authors:** Chih-Chieh Yu, Kuan-Hung Yeh, Hung-Yu Chang, Ying-Chieh Liao, Li-Ting Ho, Ting-Chun Huang, Lian-Yu Lin, Ju-Yi Chen

**Affiliations:** a Division of Cardiology, Department of Internal Medicine, National Taiwan University Hospital, Taipei, Taiwan; b Division of Cardiology, Department of Internal Medicine, Taipei Tzu Chi Hospital, Buddhist Tzu Chi Medical Foundation, New Taipei City, Taiwan; c School of Medicine, Tzu Chi University, Hualien City, Taiwan; d Heart Center, Cheng Hsin General Hospital, Taipei, Taiwan; e School of Medicine, College of Medicine, National Yang Ming Chiao Tung University, Taipei, Taiwan; f Division of Cardiology, Department of Internal Medicine, Changhua Christian Hospital, Changhua, Taiwan; g Department of Post Baccalaureate Medicine, College of Medicine, National Chung Hsing University, Taichung, Taiwan; h Division of Cardiology, Department of Internal Medicine, National Cheng Kung University Hospital, College of Medicine, National Cheng Kung University, Tainan, Taiwan; i Department of Internal Medicine, College of Medicine, National Taiwan University, Taipei, Taiwan

**Keywords:** cardiac implantable electronic devices, implantable cardioverter defibrillator, infection, pacemaker

Although cardiac implantable electronic device infections are rare, they are associated with high mortality.^1^^,^^2^ In response, clinicians often administer antibiotics more liberally than necessary, increasing costs and promoting antibiotic resistance. Preprocedural antibiotics are recommended to reduce infection risk, but extending antibiotic use beyond 24 hours postprocedure has shown no additional benefits.^3^^,^^4^ The majority of procedures are considered low risk, with infection rates expected to be minimal, and guidelines suggest that postoperative antibiotics are not necessary.^5^^,^^6^

Nevertheless, previous studies have reported varying effects of infection prevention strategies between different regions, and randomized clinical trials (RCTs) are lacking.^7^ To address this, we conducted a multicenter RCT in Taiwan to evaluate the cardiac implantable electronic device infection rate among Taiwanese patients and to provide evidence to inform future antibiotic guidelines.

This is a prospective RCT, investigating post-procedure antibiotic prophylaxis for cardiac electrical device implantation (ABxFREE [Post-procedure Antibiotic Prophylaxis for Cardiac Electrical Device Implantation: ABxFREE Study), registered at ClinicalTrials.gov (NCT04146883). This study complied with the Declaration of Helsinki and was approved by the Institutional Review Boards at National Cheng Kung University Hospital (B-ER-108-130), National Taiwan University Hospital (201906017RIND), Taipei Tzu Chi Hospital (08-X-087), Cheng Hsin General Hospital ([716]108-32), and Changhua Christian Hospital (200602).

Between August 2019 and December 2022, patients scheduled for pacemaker or implantable cardioverter-defibrillator (ICD) implantation or replacement were consecutively enrolled. Exclusion criteria included hospitalization exceeding 7 days, immunocompromised conditions (eg, end-stage renal disease requiring dialysis, chronic steroid therapy, unhealed wounds), cardiac resynchronization therapy, or undergoing His bundle pacing (HBP) or left bundle branch area pacing (LBBaP).

All patients received intravenous prophylaxis with either 1 g cephazolin or 600 mg clindamycin within 1 hour prior to the initiation of the device implantation procedure. On successful implantation, patients were randomized to either no postprocedural prophylactic antibiotics (PPA) (study group) or standard PPA (the control group), which received 1 g cephazolin intravenously every 8 hours for a total of 3 doses, followed by 3 days of oral antibiotics. The primary outcome was defined as pacemaker infection, which includes pocket erosion or discharge requiring hospitalization.

Continuous variables were assessed for normality using the Shapiro-Wilk test. Because data were not normally distributed, continuous variables are presented as medians (Q1-Q3). Categorical variables are expressed as frequencies and percentages. Comparisons between groups were performed using the Mann-Whitney *U* test for continuous variables and the chi-square or Fisher exact test for categorical variables. *P* < 0.05 was considered statistically significant. The original design aimed for a total sample size of 800 patients, with 400 patients allocated to each arm. This calculation was based on a noninferiority design, assuming infection rates of 2.0% (treatment) and 0.9% (control),^4^^,^^8^ a 1.0% noninferiority margin, α of 0.05, 80% power, and no dropout, yielding 800 participants in total.

A total of 296 patients were enrolled and successfully randomized. In the control group, 1 patient had suture dislodgement at the first follow-up. The operator placed an additional suture and prescribed oral cephalexin for an additional 7 days. This patient was subsequently excluded from the analysis due to a protocol violation. Additionally, 8 patients in the study group and 6 patients in the control group experienced hospitalizations exceeding 7 days. To control for the potential influence of other comorbidities, these 15 patients were also excluded from the final analysis.

A total of 148 patients were enrolled in the study group, and 133 in the control group. The median age was 76.0 years [Q1-Q3: 68.0-84.0 years], with an equal distribution of men (50.2%) and women (49.8%). All parameters were evenly distributed between the 2 groups. The major indication for device implantation was sick sinus syndrome. ICD implantation accounted for 47 cases (26.8%). The procedure times for new implantation and replacements were 54.0 minutes [Q1-Q3: 45.0-62.0 minutes] and 31.4 minutes [Q1-Q3: 24.0-44.5 minutes], respectively. There were no significant differences in other procedural aspects between the 2 groups. The mean follow-up was 1,056.7 ± 381.6 days. Acute complications occurred in 6 patients (2.2%), with 3 cases of pneumothorax and 3 cases of hematoma. The overall infection rate was low, affecting 3 patients (1.1%): 1 patient (0.7%) in the study group and 2 patients (1.5%) in the control group. There was no significant difference in infection rates between the 2 groups. All infection events occurred within the first 6 months of follow-up. No significant difference was observed in time to infection between the 2 groups. The post hoc analysis identified an increased risk of infection associated with younger age, occurrence of pneumothorax, and ICD implantation. However, these associations became statistically insignificant after multivariate adjustment.

This study is the first multicenter, prospective RCT conducted in Asia, initiated in 2019. The original target enrollment was 800 patients, based on an anticipated infection rate of 1.36% from a previous observational study.^8^ This target was determined through a power calculation aiming to detect a significant difference in infection rates between groups. Due to the early termination and reduced sample size, the study was underpowered to draw definitive conclusions. However, the study was ultimately terminated early due to several challenges. First, at the time of study planning and site initiation, HBP and LBBaP were undergoing early clinical exploration. At that time, these procedures were associated with longer procedures times and uncertain clinical benefits. To minimize heterogeneity and preserve the clarity of antibiotic efficacy assessment, we excluded patients undergoing HBP or LBBaP. However, as LBBaP gained more supporting evidence and was increasingly adopted into routine practice, this exclusion criterion became more restrictive and progressively hindered patient recruitment. Second, the COVID-19 pandemic significantly disrupted patient recruitment across all participating centers. Despite these limitations, the study demonstrated a low infection rate (0.7% of the study group, 1.5% of the control group), comparable to that observed in previous observational cohorts. Notably, 2 of the 3 infection cases occurred in patients who underwent ICD implantation for ventricular tachycardia and heart failure.

From an Asian perspective, this population remains of particular interest, as the relatively smaller body habitus of Asian individuals may render the larger ICD generator a proportionally greater foreign body burden compared to Western populations. However, whether this increased bulk translates into a greater clinical impact in Asian patients requires further investigation. In this study, we deliberately excluded procedures with expected longer procedure time, such as cardiac resynchronization therapy, HBP, and LBBaP, to reduce procedural variability. Although ICD and permanent pacemaker implantations are comparable in terms of procedure time, the relatively larger size of the ICD generator may confer a higher risk of infection. Due to the limited number of cases, this study was underpowered to evaluate the potential benefit of PPA in patients receiving ICDs. Future studies with larger, adequately powered cohorts, focusing on higher-risk populations, including ICD implantation, conduction system pacing, or cardiac resynchronization therapy, particularly among thinner Asian individuals, are warranted to define PPA’s role in preventing infections.

The 5 participating hospitals included 4 medical centers (geographically distributed) and 1 regional hospital to enhance the generalizability. Whereas randomization was stratified by center to balance enrollment, no additional statistical adjustments for center heterogeneity were performed due to the limited events.

The relationship between age and the risk of cardiac implantable electronic device–related infection has been inconsistently reported in previous studies.^9^^,^^10^ Although these findings appear contradictory, they underscore the complexity of the relationship between age and infection risk and highlight the need for further investigation.

## Funding Support and Author Disclosures

The contents of this manuscript are the authors’ sole responsibility, and the funding sources were not involved in any study processes. All authors take responsibility for all aspects of the reliability and freedom from bias of the data presented and its interpretation. The authors would like to thank the National Science and Technology Council of the Republic of China, Taiwan, for financially supporting this research under contract NSTC 114-2640-E-006-010. The authors have reported that they have no relationships relevant to the contents of this paper to disclose.
